# Anorectal angle at rest predicting successful sacral nerve stimulation in idiopathic fecal incontinence—a cohort analysis

**DOI:** 10.1007/s00384-020-03720-w

**Published:** 2020-08-18

**Authors:** Cathérine T. Kollmann, Elise B. Pretzsch, Andreas Kunz, Christoph Isbert, Katica Krajinovic, Joachim Reibetanz, Mia Kim

**Affiliations:** 1 Department of General, Visceral, Transplant, Vascular and Pediatric Surgery, University Hospital Wurzburg, Oberduerrbacher Strasse 6, 97080 Wurzburg, Germany; 2 Diagnostic and Interventional Radiology Institute, University Hospital Wurzburg, Oberduerrbacher Strasse 6, 97080 Wurzburg, Germany; 3 Department of General and Visceral Surgery, Amalie Sieveking Hospital, Haselkamp 33, Hamburg, Germany

**Keywords:** Sacral nerve stimulation, Idiopathic fecal incontinence, Sacral neuromodulation, Anorectal angle

## Abstract

**Purpose:**

Sacral nerve stimulation is an effective treatment for patients suffering from fecal incontinence. However, less is known about predictors of success before stimulation. The purpose of this study was to identify predictors of successful sacral nerve stimulation in patients with idiopathic fecal incontinence.

**Methods:**

Consecutive female patients, receiving peripheral nerve evaluation and sacral nerve stimulation between September 2008 and October 2014, suffering from idiopathic fecal incontinence were included in this study. Preoperative patient’s characteristics, anal manometry, and defecography results were collected prospectively and investigated by retrospective analysis. Main outcome measures were independent predictors of treatment success after sacral nerve stimulation.

**Results:**

From, all in all, 54 patients suffering from idiopathic fecal incontinence receiving peripheral nerve evaluation, favorable outcome was achieved in 23 of 30 patients after sacral nerve stimulation (per protocol 76.7%; intention to treat 42.6%). From all analyzed characteristics, wide anorectal angle at rest in preoperative defecography was the only independent predictor of favorable outcome in multivariate analysis (favorable 134.1 ± 13.9° versus unfavorable 118.6 ± 17.1°).

**Conclusions:**

Anorectal angle at rest in preoperative defecography might present a predictor of outcome after sacral nerve stimulation in patients with idiopathic fecal incontinence.

## Introduction

Fecal incontinence (FI) is a debilitating disorder that substantially diminishes quality of life as many patients are afflicted by personal consequences such as loss of independence, lowered self-esteem, and social isolation. Prevalence is 11.2% (range 8.3–13.2%) in high-quality studies, but due to social implications, the estimated number might be crucially higher [1, 2]. Sacral nerve stimulation (SNS) represents the currently preferred surgical procedure in the treatment of refractory FI, as it has shown to reduce incontinence symptoms and increase quality of life considerably and more effectively than medical treatment [3–5]. Several studies have demonstrated a short-term beneficial outcome of more than 80% after implantation [6, 7] and a preservation of treatment success over 5 years in 55.6–71.3% in per-protocol analyses [8, 9]. However, high rates of adverse events in up to 85% of patients after SNS have been described with loss and lack of efficacy as the most common events, occurring in 50% and 39% respectively [10]. Currently, less is known about pre-treatment patient’s characteristics, including demographic data, preoperative anal manometry, and defecography, and their contribution to the subsequent success of SNS.

The aim of the study was to investigate preoperative factors predicting success of SNS implantation in patients with idiopathic FI, refractory to conservative treatment. Therefore, patient’s demographics, anal manometry, and defecography before SNS were analyzed.

## Methods

Data from a prospectively collected database of consecutive patients who underwent peripheral nerve evaluation (PNE) and/or SNS device implantation from September 2008 to October 2014 at the University Hospital Wurzburg, Germany, were reviewed retrospectively. Collected data comprised patient’s demographic data, preoperative investigations, and operative details.

Preoperative investigations included physical examination, anorectal manometry, endoanal ultrasound, Cleveland Clinic Incontinence Score (CCIS), and defecography. Inclusion criteria were completion of PNE and SNS, female gender, and age of ≥ 18 years. In order to preclude heterogeneity of the study cohort and intermixing different causes of FI, only patients with idiopathic FI were included. Patients were classified as having idiopathic FI if there was no history of disorders of the central nervous system such as stroke or incomplete spinal cord injury, no diabetic neuropathy, and no larger sphincter injury (> 45°) or any defect of the pelvic floor in endoanal ultrasound [11]. Further exclusion criteria were low anterior resection syndrome (LARS) and male gender for obtaining a homogeneous patient population. SNS was performed as a two-stage procedure with a quadripolar lead and stimulator implantation after favorable unipolar PNE. Follow-up was conducted in our outpatient clinic.

Primary endpoint of the analysis was favorable and unfavorable functional results after SNS. A favorable outcome was defined as previously described, if the patient reported a benefit from the intervention and did not consider ending the stimulation [8]. If the patient described lack or loss of efficacy in spite of device reprogramming, wished to end SNS treatment, or presented with a CCIS ≥ 10, the outcome was determined unfavorable, including deactivation or explantation of the device due to lack or loss of efficacy. Lack of efficacy was defined as if the patient experienced no therapeutic benefit after SNS implantation and loss of efficacy in case of deterioration after initial improvement [8]. Patients with device explantation due to postoperative infection were excluded from further analysis, as infection was not considered to be associated with pre-treatment patient’s manometry or defecography results.

Due to its retrospective character, no approval by the local ethics committee was needed for the presented analysis, which was confirmed by local ethics committee (Number 20190821 01). All patients gave informed consent.

### Sacral nerve stimulation and defecography

PNE and SNS were performed as described before by our group [12]. Briefly, electrodes were inserted under general anesthesia into the sacral foramina S3 or S4 according to the best motor response, demonstrated by intraoperative contraction of the pelvic floor and the anal sphincter at the lowest voltage. Temporary monopolar electrodes (3059; Medtronic, Minneapolis, MN, USA) were used in the initial procedure (PNE). The external stimulation was initiated on the first postoperative day continuously with a pulse width of 210 μs and a frequency of 21 Hz. The stimulation amplitude was adjusted to patient’s subsensory threshold. During the screening phase, the patients were asked to complete a diary to record function. After 2 weeks, an adequate response was defined as a 50% reduction of incontinence episodes per week. If criteria were fulfilled, permanent SNS was advised. Therefore, a quadripolar electrode (InterStim® 3889; Medtronic) was implanted, and the implantable pulse generator (InterStim® 3023 or InterStim® 3058; Medtronic) was placed subcutaneously in the gluteal region.

Defecography was carried out in a standardized manner as described before by our group and others [12]. In brief, preparation included rectal, vaginal, vesical, and small-bowel instillation of contrast medium. The examination was conducted in an upright sitting position using X-ray imaging in a latero-lateral projection at rest, at squeeze, and during evacuation. Pelvic floor descent was existent, when the anorectal junction at rest was positioned lower than 3 cm beneath the pubococcygeal line (PCL), which was determined as the line between the inferior margin of the pubic symphysis and the caudal tip of the coccyx. Bulging of the anterior rectal wall of 2 cm or more in the anterior–posterior dimension during defecation was described as rectocele. An intussusception was defined as a circumferential infolding of the rectal wall during defecation, a descent of the bladder floor beneath the PCL was specified as vesicocele, and the anorectal angle was measured between the longitudinal axis of the anal canal and the posterior distal rectal wall on the impression of the puborectalis muscle and the tangential of the posterior rectal wall during rest, squeezing, and defecation [13].

### Statistical analysis

Statistical analysis was performed using GraphPad Prism 8.1.2 (GraphPad Software, San Diego, CA, USA) and Excel plus XLMiner Analysis ToolPak (Frontline Systems Inc.). Each set of continuous data was tested for distribution by using the Shapiro–Wilk normality test. If the test for normality confirmed a Gaussian distribution, further analysis was performed using an unpaired *t* test with Welch’s correction. Non-parametric data was compared with the Mann–Whitney *U* test. Contingency analysis for frequency of categorical data was performed using Fisher’s exact test and the chi-square test for trend. Data are presented as mean ± standard deviation (SD), if not stated otherwise. A multivariate logistic regression analysis was carried out to identify possible predictive factors for favorable or unfavorable SNS. The results are reported as 95% confidence interval (95% CI) and odds ratio (OR). Results were considered statistically significant, if the *p* value was ≤ 0.05.

## Results

### Cohort characteristics

Eighty-five patients underwent PNE between September 2008 and October 2014. Five patients refused further surgical treatment. A total of 26 patients were excluded from analysis: eleven patients suffered from traumatic FI, eleven patients from neuropathic FI, three patients from LARS, and one male patient was excluded. From fifty-four included patients, thirty-five (64.8%) reported a favorable outcome after PNE and were eligible for SNS. Reasons for an unfavorable outcome of PNE were lack of efficacy in sixteen patients (84.2%), lead dislocation in two patients (10.5%), and inadequate motor response during surgery in one patient (5.3%). All thirty-five patients with favorable PNE resulted in implantation of a permanent stimulation device (InterStim® I 3023 in 27 patients (77.1%), InterStim® II 3058 in 8 patients (22.9%)). The permanent lead was positioned at S3 in thirty patients (85.7%) and at S4 in five patients (14.3%).

**Fig. 1:**
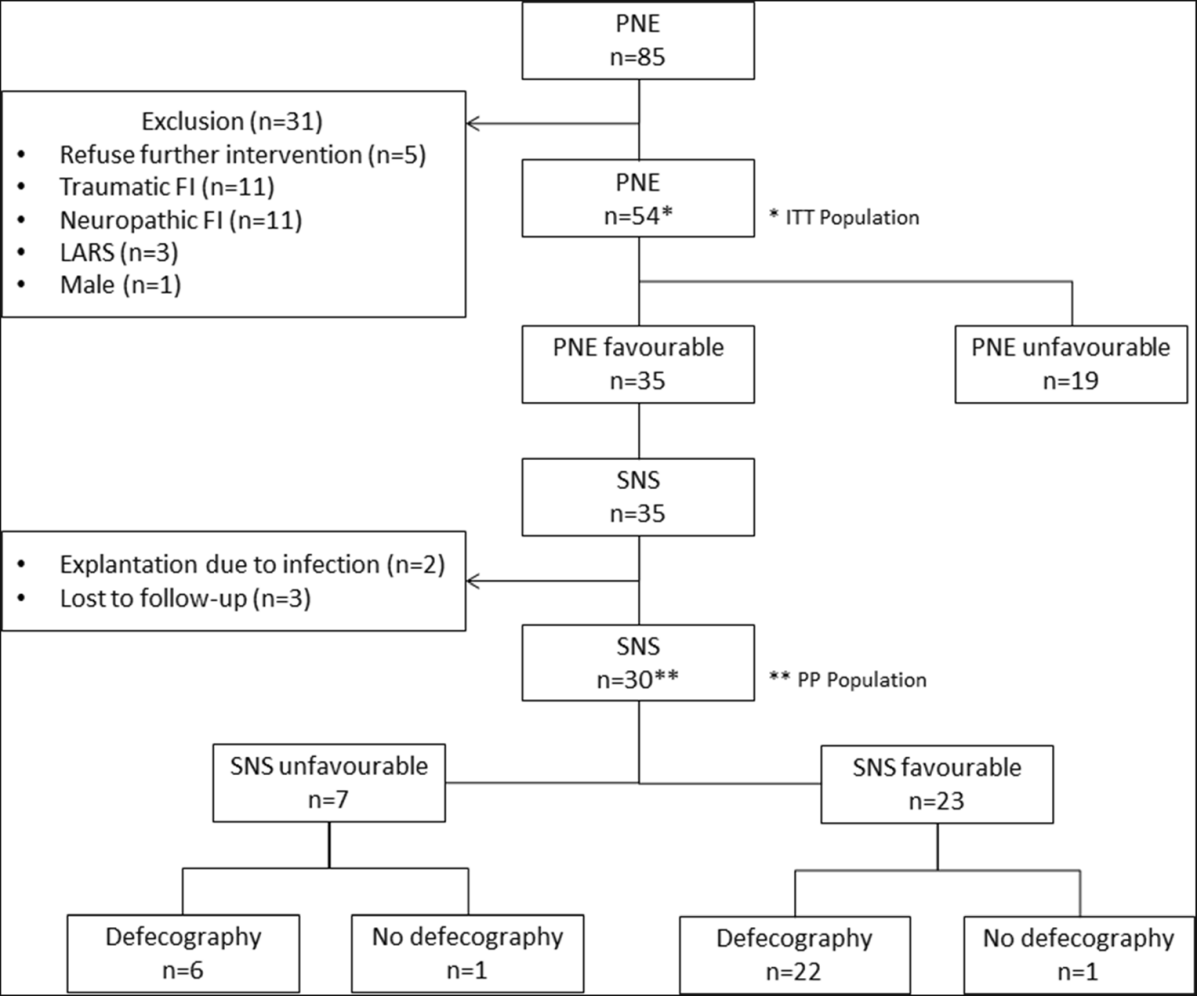
Flowchart study cohort. PNE, peripheral nerve evaluation; FI, fecal incontinence; LARS, low anterior rectal resection syndrome; SNS, sacral nerve stimulation; ITT, intention to treat; PP, per protocol

Two patients (5.7%) were excluded from functional analysis, having the SNS explanted due to postoperative infection, and three patients (8.6%) were lost to follow-up. All in all, 30 patients were eligible for assessment of functional outcome with 23 patients (per protocol (PP) 76.7% with 23 of 30 eligible patients, intention to treat (ITT) 42.6% with 23 of 54 included patients) benefitting from favorable and seven patients (PP 23.3%, ITT 13.0%) with unfavorable outcome after median follow-up of 44.5 months (range 6–109) (Fig. 1). Reasons for unsuccessful treatment after SNS were lack of efficacy in one patient (14.3%) and loss of efficacy in six patients (85.7%). Within the cohort with favorable outcome, adverse events were recorded in 16 patients (69.6%). This included lack of efficacy in one patient (4.4%), loss of efficacy in 11 patients (47.8%), pain in six patients (23.1%), infection in two patients (8.7%), and lead dislocation in one patient (4.4%), with some patients reporting more than one adverse event. Lack or loss of function was resolved by adjusting the pulse generator, in some cases multiple times. Pain was treated successfully by analgesics and infection by antibiotics in both cases. The dislocated electrode was replaced surgically.

Patients presented with a mean CCIS of 13.9 ± 4.0 before surgery. Twenty-three underwent hysterectomy (76.7%) and eleven patients stapled transanal rectal resection (STARR) in their previous history (36.7%), and most patients were multiparous (*n* = 18; 60.0%). Manometry results showed a mean resting pressure of 22.6 ± 13.6 mmHg, an average squeezing pressure of 56.6 ± 25.8 mmHg, and a coughing pressure of 52.6 ± 25.0 mmHg before SNS. Table 1 shows patients’ demographics. Neither demographic nor clinical data nor manometry results differed between the groups of favorable and unfavorable patient’s outcome (Table 1).

**Table 1:** Cohort demographics of patients with idiopathic fecal incontinence

	All patients (*n* = 30)	Favorable SNS (*n* = 23)	Unfavorable SNS (*n* = 7)	*p*
Age, years	71.8 ± 8.1	71.7 ± 8.6	72.1 ± 6.7	0.88
Type of FI, *n* (%)				
Urge	20 (66.7)	16 (69.6)	4 (57.1)	0.84
Passive	3 (10.0)	3 (13.0)	0 (0)	
Combined	5 (16.7)	4 (17.4)	1 (14.3)	
No data	3 (10.0)	1 (4.4)	2 (28.6)	-
History of FI, months (median, range)	34.0 (6–720)	36.0 (6–480)	24.0 (7–720)	0.88
Previous operation, *n* (%)				
Hysterectomy	23 (76.7)	17 (73.9)	6 (85.7)	> 0.99
STARR	11 (36.7)	7 (30.4)	4 (57.1)	0.37
Childbirth				
Multipara	18 (60.0)	14 (60.9)	4 (57.1)	0.27
Unipara	5 (16.7)	4 (17.4)	1 (14.3)	
Nullipara	1 (3.3)	0 (0)	1 (14.3)	
No data	6 (20.0)	5 (21.7)	1 (14.3)	-
CCIS, pts	13.9 ± 4.0	14.1 ± 3.9	13.1 ± 4.5	0.63
Manometry pressure, mmHg				
Resting	22.6 ± 13.6	23.5 ± 14.7	19.6 ± 9.7	0.42
Squeezing	56.6 ± 25.8	53.3 ± 24.7	67.6 ± 28.3	0.26
Coughing	52.6 ± 25.0	52.0 ± 23.3	54.6 ± 1.8	0.85
Length of follow-up, months (median, range)	44.5 (6–109)	47.0 (6–109)	36.0 (17–87)	0.92
Defecography, *n* (%)				
Yes	28 (93.3)	22 (95.7)	6 (85.7)	0.42
No	2 (6.7)	1 (4.4)	1 (14.3)	

*SNS*, sacral nerve stimulation; *FI*, fecal incontinence; *STARR*, stapled transanal rectal resection; *CCIS*, Cleveland Clinic Incontinence Score

Data is presented as mean ± standard deviation, if not stated otherwise

### Defecography

Of thirty patients receiving SNS, defecography was available in twenty-eight (93.3%). The number of patients with pelvic floor descent at rest did not differ between favorable and unfavorable outcome groups (13/22, 59.1% and 3/6, 50% respectively; *p* > 0.99), neither did the number of patients with rectocele (12/22, 54.6% versus 2/6, 33.3%; *p* = 0.65). Also, intussusception did not differ significantly between patients with favorable and unfavorable outcome (18/22, 81.8% and 4/6, 66.7% respectively; *p* = 0.58), as well as existence of vesicocele (11/22, 50% versus 2/6, 33.3%; *p* = 0.66) or enterocele (5/22, 22.7% versus 0/6, 0%; *p* = 0.55) (Table 2).

**Table 2:** Results from preoperative defecography in patients with idiopathic fecal incontinence

	Favorable SNS (*n* = 22)	Unfavorable SNS (*n* = 6)	*p* univariate
Pelvic floor descent, *n* (%)	13 (59.1)	3 (50.0)	> 0.99
Rectocele, *n* (%)	12 (54.6)	2 (33.3)	0.65
Intussusception, *n* (%)	18 (81.8)	4 (66.7)	0.58
Vesicocele, *n* (%)	11 (50.0)	2 (33.3)	0.66
Enterocele, *n* (%)	5 (22.7)	0 (0)	0.55
Anorectal angle at rest, degree	134.1 ± 13.9	118.6 ± 17.1	0.03
Anorectal angle at squeeze, degree	126.2 ± 18.8	106.3 ± 24.1	0.07
Anorectal angle during Valsalva, degree	135.2 ± 15.9	126.2 ± 13.0	0.19

*SNS*, sacral nerve stimulation

Data is presented as mean ± standard deviation, if not stated otherwise

The anorectal angle at rest was the only parameter in univariate analysis that was significantly different between the groups with a mean angle of 134.1 ± 13.9° in patients with favorable versus 118.6 ± 17.1° in patients with unfavorable outcome (*p* = 0.03; *R*^2^ = 0.17). There was no significant difference in anorectal angles during squeeze (favorable 126.2 ± 18.8°; unfavorable 106.3 ± 24.1°; *p* = 0.07) or Valsalva maneuver (favorable 135.2 ± 15.9°; unfavorable 126.2 ± 13°; *p* = 0.19) (Table 2).

A multivariate analysis, including the anorectal angle at rest as the only significant factor in univariate analysis, and intussusception as described as a potential predictor of success in literature previously were analyzed [14]. A wide anorectal angle at rest was the only independent parameter predicting favorable outcome in patients with idiopathic FI in multivariate analysis (OR 1.06, 95% CI 1.0002–1.13; *p* = 0.049; *R*^2^ = 0.17), while the presence of intussusception was not (OR 2.04, 95% CI 0.23–18.26; *p* = 0.52; *R*^2^ = 0.17).

## Discussion and conclusions

In spite of the widespread application of SNS in the surgical treatment algorithm of refractory FI, its neurophysiological mechanisms of action remain still unclear. Our data show for the first time the role of the anorectal angle at rest and therefore the relevance of the puborectalis muscle for the effectiveness of SNS treatment as a potential preoperative predictor of success in patients suffering from idiopathic FI.

Success rates of test stimulation and of permanent stimulation of 65% and 74.3% respectively in the per-protocol analysis in our study were in line with previously published data [6–9]. The limited success in the intention-to-treat analysis of 42% after SNS underlines the need for improving patient selection, as the exact mechanism of SNS remains to be elucidated. Moreover, application of SNS is costly, especially considering the high rates of adverse events, requiring explantation in 15–25% of patients, with 38% suffering from lack or loss of efficacy and 13% from pain in long-term follow-up [3, 15]. The number of patients requiring any surgical reintervention is even higher with 29%, of which most accounted for lead-associated problems, causing lack or loss of efficacy in most cases [16]. Therefore, there is an urgent need for anticipating success of SNS before implantation in order to select patients and to give advice for shared decision-making.

In our cohort, from all preoperatively analyzed patient’s characteristics, including defecography, the only predictive factor for favorable functional outcome was an increased anorectal angle at rest in defecography before surgery. Until now, preoperative characteristics, predicting success after SNS, are scarce, especially regarding defecography findings.

Several studies analyzed predictors of success, but results were contradictory, and no concordant clinical predictor was found: In a single-center study of 81 analyzed patients suffering from any kind of FI, success of SNS was not influenced by any baseline demographics, such as gender, age, or duration of symptoms, comparable to our results. Low amplitude to achieve a satisfactory motor response predicted successful and repeat PNE testing no successful stimulation. Moreover, evidence of internal and/or external anal sphincter damage was associated with a worse functional outcome than in the absence of sphincter damage [17]. A study by Maeda and coworkers, evaluating 141 SNS patients, could show that increasing age was an independent risk factor for treatment failure in FI patients, and improvement of urge incontinence episodes during PNE as well as improvement of incontinence 6 months after SNS predicted long-term treatment success [8]. While age was an independent predictor in the latter study with increasing risk with every 1-year increase in age, results of our study and of others could not demonstrate an influence of age on the outcome of SNS, which might be due to homogenous age distribution in our cohort and the smaller patient number, focusing on a subgroup of patients. Furthermore, no baseline demographics nor investigations such as anal manometry predicted functional outcome, which is compatible with our findings [8].

The presented study cohort included eleven patients, who underwent the STARR procedure before SNS implantation. Although a current systematic review showed no evidence of the STARR procedure contributing to FI on the basis of level II evidence, urge symptoms after STARR are described in 0–34%, and de novo FI in up to 9% in individual cohorts [12, 18, 19]. While small rectal diameter and increased pelvic floor descent in defecography predict FI after STARR, the effect of STARR on the outcome of SNS has not been studied yet [12].

One other study addressed the impact of defecography before surgery with special attention to the presence of high-grade internal rectal prolapse in 106 patients suffering from FI not distinguishing between the different entities of FI. The authors could show that the presence of high-grade internal rectal prolapse in defecography had a negative impact on SNS outcome. The presence of rectocele and enterocele was not associated with functional outcome, which is again compatible with our results [14]. In our study, the presence of intussusception was not a predictive factor of success in a cohort with smaller sample size, analyzing selectively patients with idiopathic FI. Due to smaller sample size, we did not subdivide into low-grade and high-grade intusussception, while Prapasrivorakul et al. did not evaluate the anorectal angle, wherefore the results of both studies are difficult to compare.

The presented results are the first demonstrating a weak puborectalis muscle as a potential predictor of success before SNS, while few studies have analyzed the postoperative effect of SNS on the anorectal angle.

We chose to define the anorectal angle as the axis of the anal canal and the distal half of the posterior wall of the rectum on the impression of the puborectalis muscle as described before [13, 20, 21]. High intra- and interobserver agreement has been attributed to this definition [20, 21]. Moreover, the “posterior” anorectal angle at rest was demonstrated to be an independent risk factor for the severity of FI with a wide anorectal angle at rest predicting more severe FI, implying an essential role of the puborectalis muscle in maintaining continence [21].

In neurophysiological studies, Matzel and coworkers could show that neurostimulation of S3 leads to a decrease of the anorectal angle in radiography, confirming the important role of the puborectalis muscle also in the mechanism of SNS [22]. These findings were supported by Uludag et al., who could demonstrate that the activated implanted pulse generator induces a decreased anorectal angle in patients with idiopathic FI. As only eleven patients were included in this series, the results did not reach statistical significance [23]. The importance of the anorectal angle and therefore of the puborectalis sling might also explain the success of SNS in patients with traumatic FI even with up to one-third of the circumference of the anal sphincter disrupted [24]. Possibly, that is why preoperative anal pressures are no predictors of favorable outcome after SNS, neither in our study nor in others [8, 23]. Our findings are in line with these previous studies, confirming the role of the puborectalis muscle as a crucial point of action in the mechanism of SNS, demonstrating that especially those with an increased anorectal angle and a weak puborectalis sling might benefit from SNS [22, 23].

However, the exact mechanism of action of SNS in FI remains poorly understood, and more complex processes than direct stimulation of the efferent motoneurons resulting in an activation of the anal sphincter can be assumed. As the latency from sacral stimulation to anal sphincter response takes longer than expected from exclusive efferent motor nerve supply, contractions seem to be triggered by afferent pathways [25]. Electromyographic analysis during SNS stimulation suggests that anal sphincter contraction is reinforced by the afferent precipitated response, and increased afferent signaling impedes descending inhibition of the pelvic contraction on a supraspinal level [26]. SNS also induces alterations in rectal sensory perception with subsequent reduced thresholds of filling sensation and urge to defecate, which might be transmitted by autonomic afferent sacral reflexes [27, 28]. Also, central modulation might contribute to the effects of SNS, significantly influencing the excitability of corticoanal pathways and the somatosensory cortex [29, 30]. As evidence arises that SNS may probably affect multiple neurophysiological pathways, from motoric and sensoric afferent modulation to central plasticity, further studies are needed for a better understanding of the underlying mechanisms.

For clinical practice, our results, indicating the exceptional role of the puborectalis muscle in predicting functional outcome of SNS, might offer potential guidance for treatment strategies and advice for shared decision-making. The results of the presented study are limited by its retrospective design and the small patient cohort, including a considerable number of patients who underwent the STARR procedure before SNS. Therefore, our results should be considered carefully, allowing only preliminary conclusions. Nonetheless, as SNS is associated with a considerable number of adverse events and surgical reinterventions, our results might pave the way for a targeted application of SNS in patients suffering from refractory FI, for whom few predictors for any surgical treatment success exist to date.

## Data Availability

Data was collected at our institution.
